# Nonreciprocal light propagation in coupled microcavities system beyond weak-excitation approximation

**DOI:** 10.1038/s41598-017-14397-7

**Published:** 2017-10-25

**Authors:** Anshou Zheng, Guangyong Zhang, Hongyun Chen, Tingting Mei, Jibing Liu

**Affiliations:** 1 School of Mathematics and Physics, China University of Geosciences, Wuhan, 430074 China; 2 Hubei Key Laboratory of Pollutant Analysis and Reuse Technology and Department of Physics, Hubei Normal University, Huangshi, 435002 China

## Abstract

We propose a scheme for nonreciprocal light propagation in two coupled cavities system, in which a two-level quantum emitter is coupled to one of the optical microcavities. For the case of parity-time (${\mathscr{P}}{\mathscr{T}}$) symmetric system (i.e., coupled active-passive cavities system), the cavity gain can significantly enhance the optical nonlinearity induced by the interaction between a quantum emitter and cavity field beyond weak-excitation approximation. The increased optical nonlinearity results in the non-lossy nonreciprocal light propagation with high isolation ratio in proper parameters range. In addition, our calculations show that nonreciprocal light propagation will not be affected by the unstable output field intensity caused by optical bistability, and we can even switch directions of nonreciprocal light propagation by appropriately adjusting the system parameters.

## Introduction

Achieving rapid development in integrated photonic circuits depends on the all-optical elements, which are essential for high-speed processing of light signals. Nonreciprocal light propagation is an indispensable common trait for some optical elements, such as optical diodes, optical isolator, circulator, etc. For example, optical diode permits the light transport in only one direction but not the opposite direction. The successful design of nonreciprocal light propagation devices relies on the breaking of time-reversal symmetry. Thus, nonreciprocal light propagation is inherently difficult, even in theory because of time-reversal symmetry of light-matter interaction^1^. Motivated by the tremendous application of nonreciprocal electrical current propagation, an immense attention has been paid to the study of nonreciprocal light propagation. As a traditional method, a material with strong magneto-optical effects (faraday rotation) is often used to break time-reversal symmetry for some optical devices^2–4^. However, unfortunately the requirement of the magneto-optical effect is the big size components and strong external magnetic fields, which are harmful for the on-chip optical nonreciprocal devices. Beyond that, one can also break time-reversal symmetry and design the nonreciprocal optical devices by time-dependent effects^5,6^, unbalanced quantum coupling^7–10^ or optical nonlinearity^11–17^. The ubiquitous optical nonlinearity in different optical systems has been extensively studied and further adopted in design of nonreciprocal light propagation devices. For example, many schemes have been reported through the nonlinearity of the waveguides, such as the second order nonlinearity *χ* ^(2)^ ^11–14^, dispersion-engineered chalcogenide^15^, Raman amplification^16^ and so on.

On the other hand, due to the high-quality factor *Q* and small mode volume *V* of optical microcavities^18–21^, it has attracted considerable interest for implementing nonreciprocal light propagation devices^22–31^. For instance, Fan *et al*. achieved the experiment of nonreciprocal light propagation with the Kerr and thermal nonlinearity in silicon microring resonators^22^. Based on the nonlinearity of an optomechanical system, some schemes of nonreciprocal behaviour have also been reported^26–29^. The strong nonlinearity required for nonreciprocal light propagation is not easy to obtain, especially for few-photon situations. Recently, some works show that the nonlinearity in coupled resonators can be greatly enhanced by introducing optical gain in one resonator of the ${\mathscr{P}}{\mathscr{T}}$-symmetric system^23–25,32,33^. And an immense attention has been attracted to ${\mathscr{P}}{\mathscr{T}}$-symmetric system which has an interesting feature that non-Hermitian Hamiltonian can still have an entirely real spectrum^34,35^. In addition, two coupled resonators can be processed as a ${\mathscr{P}}{\mathscr{T}}$-symmetric system^24,25,36–39^. More recently, a few schemes of nonreciprocal light propagation have been proposed with ${\mathscr{P}}{\mathscr{T}}$-symmetric coupled resonators system^23–25^. For example, based on the inherent nonlinearity (i.e., gain-induced nonlinearity) of the ${\mathscr{P}}{\mathscr{T}}$-symmetric system, successful experiments have been carried out for nonreciprocal light propagation with two coupled whispering-gallery-mode (WGM) microresonators^24,25^. Note that through mechanical Kerr nonlinearity, a theory scheme is also proposed for nonreciprocal phonon propagation with coupled mechanical resonators^23^. The weak mechanical Kerr nonlinearity is greatly improved by the gain in one mechanical resonator of the ${\mathscr{P}}{\mathscr{T}}$-symmetry.

Based on two-level quantum emitters coupled to waveguides or microcavities, asymmetric light transmission has been experimentally observed^40–43^. In these schemes, the breaking of the time-reversal symmetry relies on the chiral (direction dependent) light-matter interaction. For example, in^40^, the input photons from the opposite directions are in completely different polarized states. The polarized photons are coupled to the spin-polarized atom in microcavity with entirely different coupling strengths. Its chiral coupling leads to nonreciprocal behaviour. Different from these schemes, we explore the optical nonreciprocal behaviour in a system of two coupled cavities and a single quantum emitter coupled to one of the cavities. Our scheme is based on the optical nonlinearity breaking time-reversal symmetry and the optical nonlinearity is induced by a single quantum emitter coupled to a microcavity beyond weak-excitation approximation. We first consider the passive-passive case (i.e., without cavity gain, two cavities are directly coupled to each other and a quantum emitter is coupled to the first cavity). Without the cavity gain, however, the nonlinearity of the system is weak. After replacing the first cavity with a gain cavity, the system becomes the active-passive case. The nonlinearity, which is enhanced by the cavity gain, leads to the remarkable nonreciprocal effect. The scheme reported here has some important features. (i) The optical nonlinearity of the hybrid system is greatly enhanced by the cavity gain. (ii) Through adjusting parameters, one can switch between the blocking and allowing directions. For the active-passive case, one can all obtain the non-lossy transmission with high isolation ratio in allowing directions. (iii) Optical bistability or even optical multistability behaviour is often induced by optical nonlinearity, and it will lead to the instability of the output field. When the disturbance and perturbation of the system parameters are strong enough, the output field intensity will skip back and forth between the different metastable values of the optical bistability. However, via choosing proper parameters range, one can avoid the interference from the instability of output field intensity and obtain certain output intensity even for the strong disturbance of parameters.

## Results

### Theoretical model

**Figure 1:**
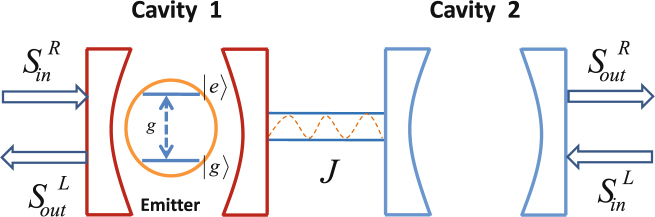
Schematic diagram of the hybrid optical system. The hybrid optical system consists of two coupled cavities and a two-level quantum emitter coupled to one of them.

We consider the setup as shown in Fig. 1, where two single-mode optical microcavities of frequencies *ω*_1(2)_ are directly coupled to each other with strength *J*. The coupling strength *J* is very sensitive to the distance between the two cavities. The optical cavities are denoted by bosonic annihilation and creation operators ${\hat{a}}_{j}$ and ${\hat{a}}_{j}^{\dagger }(j=1,\mathrm{2)}$, respectively. A two-level quantum emitter with transition frequency *ω*_*e*_ is embedded in the first (j = 1) cavity, and the cavity mode ${\hat{a}}_{1}{({\hat{a}}_{1})}^{\dagger }$ is coupled to the quantum emitter transition |*e*〉 $\iff$ |*g*〉 with the coupling strength *g*. We take the input probe field as ${S}_{in}={\varepsilon }_{p}{e}^{-i{\omega }_{p}t}$, where *ω*_*p*_ and *ε*_*p*_ is the carrier frequency and the amplitude of the probe field propagating in the waveguide. The amplitude of the input probe field *ε*_*p*_ is normalized to a photon flux at the input of the cavity and the directly related power is $P=\hslash {\omega }_{p}{\varepsilon }_{p}^{2}$. Under the rotating-wave and the electric-dipole approximation, the effective Hamiltonian of the hybrid optical system is written in the rotating frame at the frequency of the probe field *ω*_*p*_ as^44,45^

1 $$\begin{array}{rcl}{\hat{H}}_{e}^{R} & = & \hslash ({{\rm{\Delta }}}_{1}+{{\rm{\Delta }}}_{2}){\hat{\sigma }}_{ee}+\hslash {{\rm{\Delta }}}_{1}{\hat{{\rm{a}}}}_{1}^{\dagger }{\hat{a}}_{1}+\hslash {{\rm{\Delta }}}_{1}{\hat{a}}_{2}^{\dagger }{\hat{a}}_{2}+i\hslash g({\hat{a}}_{1}{\hat{\sigma }}_{eg}-{\hat{a}}_{1}^{\dagger }{\hat{\sigma }}_{ge})\\  &  & +\hslash J({\hat{a}}_{1}^{\dagger }{\hat{a}}_{2}+{\hat{a}}_{2}^{\dagger }{\hat{a}}_{1})+i\hslash \sqrt{{\kappa }_{e}}({\varepsilon }_{p}{\hat{a}}_{1}^{\dagger }-{\varepsilon }_{p}^{\ast }{\hat{a}}_{1}),\end{array}$$

and

2 $$\begin{array}{rcl}{\hat{H}}_{e}^{L} & = & \hslash ({{\rm{\Delta }}}_{1}+{{\rm{\Delta }}}_{2}){\hat{\sigma }}_{ee}+\hslash {{\rm{\Delta }}}_{1}{\hat{a}}_{1}^{\dagger }{\hat{a}}_{1}+\hslash {{\rm{\Delta }}}_{1}{\hat{a}}_{2}^{\dagger }{\hat{a}}_{2}+i\hslash g({\hat{a}}_{1}{\hat{\sigma }}_{eg}-{\hat{a}}_{1}^{\dagger }{\hat{\sigma }}_{ge})\\  &  & +\hslash J({\hat{a}}_{1}^{\dagger }{\hat{a}}_{2}+{\hat{a}}_{2}^{\dagger }{\hat{a}}_{1})+i\hslash \sqrt{{\kappa }_{e}}({\varepsilon }_{p}{\hat{a}}_{2}^{\dagger }-{\varepsilon }_{p}^{\ast }{\hat{a}}_{2}),\end{array}$$

where the Hamiltonian ${\hat{H}}_{e}^{R}$ and ${\hat{H}}_{e}^{L}$ stand for the cases of forward and backward incidence, respectively. The symbol ${\hat{\sigma }}_{ge}({\hat{\sigma }}_{eg})$ stands for the emitter descending (ascending) Pauli operator and ${\hat{\sigma }}_{ee}$ is the population operator. We assume the two cavities have the same resonant frequencies *ω*_1_ = *ω*_2_ = *ω*_*c*_. Δ_1_ = *ω*_*c*_ − *ω*_*p*_ (Δ_2_ = *ω*_*e*_ − *ω*_*c*_) is the frequency detuning of the cavity field frequency from the probe field frequency (the quantum emitter transition frequency). The coupling parameter *κ*_*e*_ describes the coupling loss rate between each cavity and the corresponding taper waveguide. The different directions of the input beam will lead to an entirely different evolution of the optical system. The phenomenon of direction-dependent evolution of the optical system can be displayed in the output field, which can be obtained through the following input-output relation^46,47^

3 $${S}_{out}^{R}=\sqrt{{\kappa }_{e}}{a}_{2}^{R},$$

4 $${S}_{out}^{L}=\sqrt{{\kappa }_{e}}{a}_{1}^{L}.$$

### Non-reciprocal light propagation

In the scheme, the strong nonlinearity of the system plays a significant role in enhancing the ability of nonreciprocity light propagation. And it is induced by the interaction between an optical cavity and an quantum emitter. In Eqs (7–14), which describe the evolution of the hybrid optical system, we focus on the nonlinear terms $g{a}_{1}^{\ast }{\sigma }_{ge}$, $g{a}_{1}{\sigma }_{ge}^{\ast }$ and −2*ga*_1_ *σ*_*z*_. With the weak-excitation approximation, these nonlinear terms are always discarded and the optical system will evolve in linear regime. In our scheme, the nonlinear terms are essential and lead to the optical bistable state, as shown in^48^. Then with the parameters in the bistable region, the considered optical system may have two different metastable output values. Once the disturbance and perturbation of the system parameters become strong, the output field will switch between the different metastale values. The uncertainty of the output field is detrimental to nonreciprocal light propagation.

**Figure 2:**
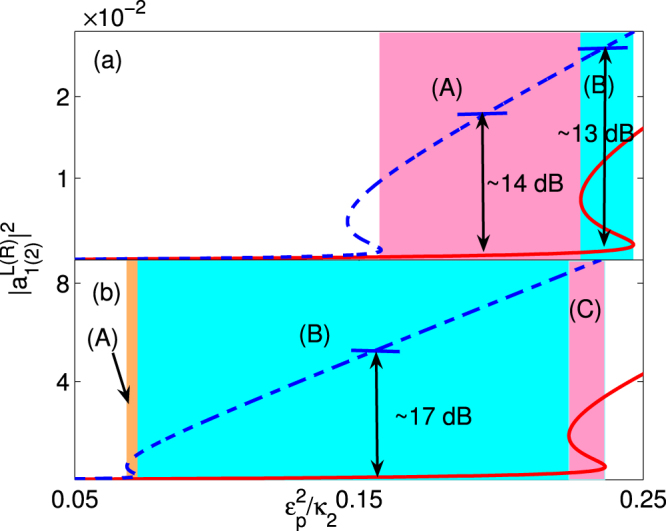
The dependence of the output field on the input field for two cases. The output field ${|{a}_{2}^{R}|}^{2}$ (red solid line) and ${|{a}_{1}^{L}|}^{2}$ (blue dashed line) vary under the input field ${\varepsilon }_{p}^{2}/{\kappa }_{2}$ with (**a**) *γ* = 0.15 *κ*_2_, *g* = 2*κ*_2_, Δ_1_ = Δ_2_ = 0, *κ*_1_ = *κ*_2_, *κ*_*e*_ = 3*κ*_2_, and *J* = 2.5*κ*_2_ for the passive-passive case; (**b**) *γ* = 0.17*κ*_2_, *g* = 2*κ*_2_, Δ_1_ = Δ_2_ = 0, *κ*_1_ = −7.4*κ*_2_, *κ*_*e*_ = 3.2*κ*_2_, and *J* = 3.8*κ*_2_ for the unbroken ${\mathscr{P}}{\mathscr{T}}$ phase (i.e., the active-passive case).

Based on the above analysis, we explore the relationship between the output and input intensity for the forward and backward propagation cases. The transmission coefficients are defined as

5 $${T}^{L(R)}={|\frac{{S}_{out}^{L(R)}}{{S}_{in}}|}^{2},$$

and the isolation ratio is

6 $$Isolation\,Ratio(dB)=10\times {\mathrm{log}}_{10}\,\frac{{T}^{L}}{{T}^{R}},$$

which quantifies the isolation performance of the system. Figure 2(a,b) correspond to the passive-passive case and active-passive case, respectively. In the color-code region of Fig. 2(a), the maximum isolation ratio is approximately 14 dB. However, in the color-code region (A) of Fig. 2(a), both the forward and backward propagation have only one stable output value. Thus, by choosing the system parameters in the color-code region (A) of Fig. 2(a), we can overcome the shortcoming of the uncertain output field intensity and also obtain the high isolation ratio. The physical mechanism underlying the nonreciprocal light transport is rooted in the cavity-quantum emitter interaction inducing nonlinearity, which significantly increases the field intensity of the first cavity. The asymmetrical coupling breaks time-reversal symmetry, which makes the nonreciprocal light transport feasible, i.e., the light transport from the cavity 2 to the cavity 1 is allowed and the light transport of the opposite direction is blocked. For the passive-passive case, due to the decay rate of the cavities and emitter, the low transmissivity (i.e.,weak output field intensity, about 30% of the input field intensity) is another obstacle to realize the nonreciprocal light transport.

In order to enhance the transmissivity, we choose the active-passive case as shown in Fig. 2(b). The gain of the first cavity greatly improves the nonlinearity and promotes the field localization in the first cavity. It allows the light propagation from the passive cavity to the active cavity and prevents the propagation in the opposite direction^24,25^. The gain-loss balance of the ${\mathscr{P}}{\mathscr{T}}$-symmetric system makes the non-lossy unidirectional light transport achievable. As shown in all the color-code areas of Fig. 2(b), we can obtain the nonreciprocal light transport with over 99% high transmissivity and about 17 dB isolation ratio. Compared with the color-code areas (A) and (C) of Fig. 2(b), the area (B) is more suitable for the unidirectional light transport without the disturbance of the uncertain output field intensity.

**Figure 3:**
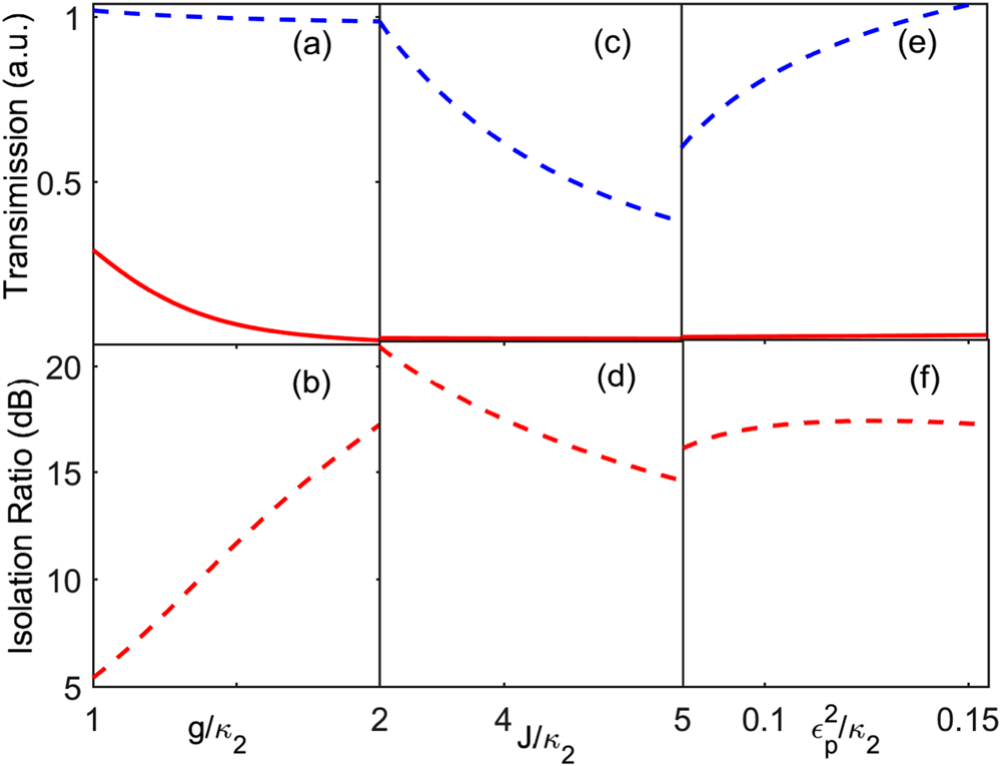
The dependence of nonreciprocal behaviour on some parameters. For the resonant case, the transmission coefficient *T* ^*R*^ (red solid line) and *T* ^*L*^ (blue dashed line), and the isolation ratio (red dashed line) varies under the different values of (**a**,**b**) the cavity-quantum emitter coupling strength *g*/*κ*_2_ with *J* = 3.8*κ*_2_, and ${\varepsilon }_{p}=0.3573\sqrt{{\kappa }_{2}}$; (**c**,**d**) the cavity-cavity coupling strength *J*/*κ*_2_ with ${\varepsilon }_{p}=0.3573\sqrt{{\kappa }_{2}}$, and *g* = 2*κ*_2_; (**e**,**f**) The input field ${\varepsilon }_{p}^{2}/{\kappa }_{2}$ with *g* = 2*κ*_2_, and *J* = 3.8*κ*_2_. The other system parameters are chosen as *γ* = 0.17*κ*_2_, *κ*_1_ = −7.4*κ*_2_, and *κ*_*e*_ = 3.2*κ*_2_, respectively.

**Figure 4:**
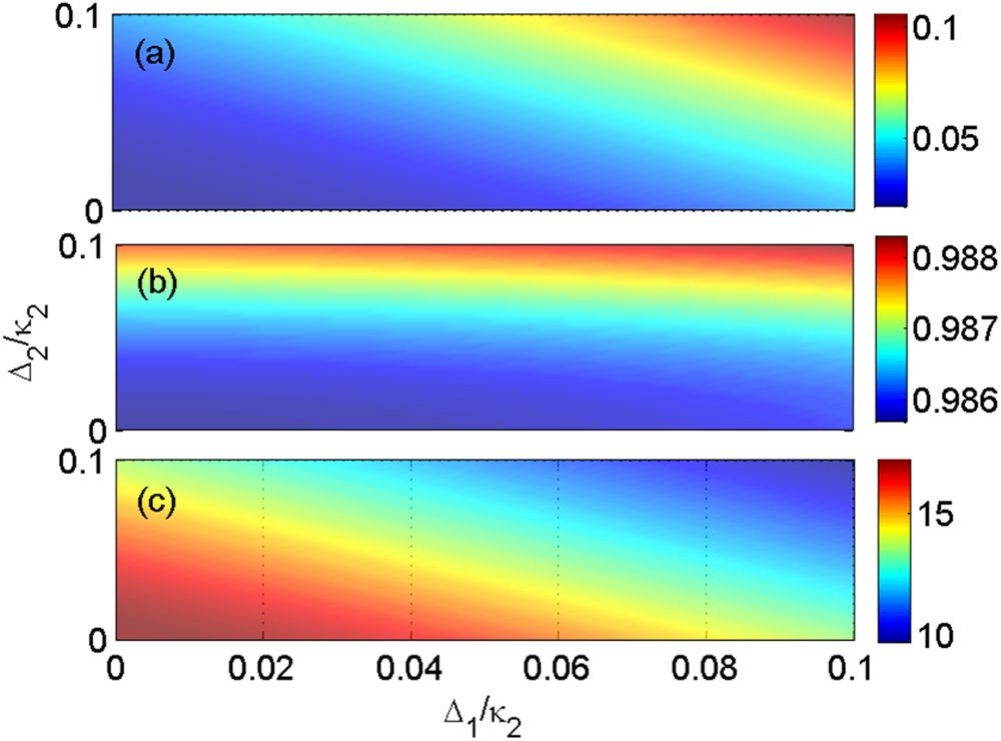
The dependence of nonreciprocal behaviour on frequency detunings. For the off-resonant case, the transmission coefficients (**a**) *T* ^*L*^ and (**b**) *T* ^*R*^, and (**c**) isolation ratio vary with frequency detunings Δ_1_/*κ*_2_ and Δ_2_/*κ*_2_. The other system parameters are chosen as *γ* = 0.17*κ*_2_, *κ*_1_ = −7.4*κ*_2_, *κ*_*e*_ = 3.2*κ*_2_, *J* = 0.8*κ*_2_, ${\varepsilon }_{p}=0.3573\sqrt{{\kappa }_{2}}$, and *g* = 2*κ*_2_, respectively.

With respect to the ${\mathscr{P}}{\mathscr{T}}$-symmetric system, we analyze the effect of varying the parameters on the unidirectional light transport in detail. When *g* is close to zero, the linear system allows the non-lossy light propagation in both directions with the help of gain of the first cavity, but the isolation ratio declines sharply. With the growing coupling strength *g*, the nonlinearity of the system increases and is greatly enhanced by the gain of the first cavity. The large nonlinearity in the first cavity (i.e., the active cavity) promotes the field localization in the active cavity and breaks time-reversal symmetry of the considered optical system. As a result, the non-lossy light propagation in the backward direction is almost unaffected and the opposite direction propagation is blocked completely. As shown in Fig. 3(b), when *g* approaches 2*κ*_2_, the isolation ratio is about 17 dB. Figure 3(c,d) show the influence of *J* on the unidirectional light propagation. The cavity-cavity coupling *J* and the cavity-quantum emitter coupling *g* compete for the input field of the system. When the cavity-quantum emitter coupling *J* is in the commanding position, the nonlinearity of system decreases sharply. Thus, the light transmissivity in the backward direction reduces with the decreasing of cavity-cavity coupling, and the isolation ratio goes down as well. From Fig. 3(e,f), we can see that the effect of the change of the input field intensity on the unidirectional light propagation can be neglected. In addition, the frequency detunings have a different influence from the cavity-cavity coupling on the present scheme. As the frequency detuning increases, the nonlinearity of the system decreases. Figure 4 shows the concrete effect of the frequency detunings on the unidirectional light propagation.

The large nonlinearity is essential to the present scheme. The nonlinearity leads to the field localization in the nonlinear cavity. Thus, the forward direction is forbidden and the other direction is allowed. For the passive-passive case, the transmissivity of the input field is low although one can get the high Isolation Ratio for the weak nonlinearity. For the active-passive case, however, the enhanced nonlinearity greatly improves the transmittance and increases the Isolation Ratio at the same time. The above analysis shows that some parameters are critical to our scheme. For instance, the nonlinearity increases with the coupling strength *g*, whereas decreases with the frequency detuning. The cavity-cavity coupling *J* is another key parameter of the system, which has an important effect on the system nonlinearity. The system is in unbroken ${\mathscr{P}}{\mathscr{T}}$-symmetric phase and the field localization effect is permitted to continue when *J* is big enough. Once the system is at phase transition point or the broken phase transition as the coupling *J* decreases, the flow of the power is suppressed from the passive cavity into the active cavity. The effect of field localization will gradually disappear and the optical system will be in the (A) region of Fig. 2(b).

### Direction-exchange

**Figure 5:**
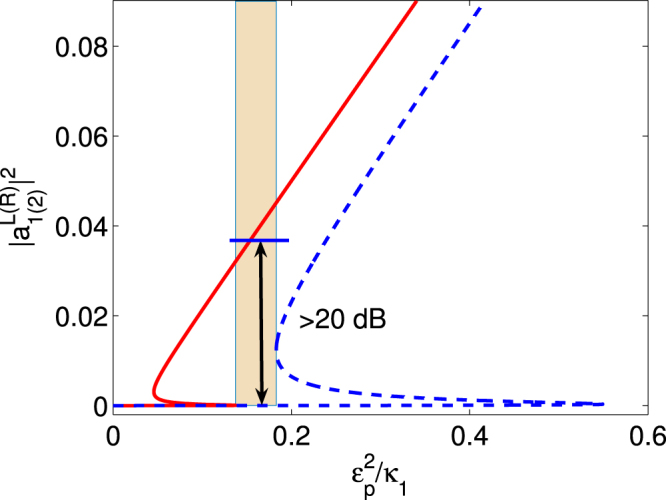
The dependence of the output field on the input field for exchange-directions. ${|{a}_{1}^{L}|}^{2}$ (blue dashed line) and ${|{a}_{2}^{R}|}^{2}$ (red solid line) vary with the input field ${\varepsilon }_{p}^{2}/{\kappa }_{2}$ for the resonant case. The parameters are *γ* = 0.1*κ*_1_, *g* = 2*κ*_1_, *κ*_2_ = −9*κ*_1_, *κ*_*e*_ = 4*κ*_1_, and *J* = 1.25*κ*_1_.

We describe an interesting feature of the optical system, i.e., the allowed direction of the unidirectional light propagation can be reversed by adjusting the system parameters. On the above analysis, the light propagation in backward direction is allowed and the opposite direction is blocked. If only the second cavity is pumped as a gain cavity, the allowed direction of the optical nonreciprocity will exchange with the blocked direction. The optical nonlinearity is transferred to the cavity with gain. As shown in Fig. 5, one can obtain the nonreciprocity with over 20 dB isolation ratio. The similar situations have been considered with different nonlinearities in coupled double-cavity system, including gain-saturation nonlinearity^24,25^ and mechanical nonlinearity^23^. In these situations, the nonlinearity is transferred from the passive cavity to the one with gain and the transferred nonlinearity leads to the field localization in the gain cavity. Compared with the above model, the transmissivity is relatively low even with the smaller decay rate *γ* = 0.1*κ*_1_ because of the transferred process.

## Discussion

In light of the recent experiment^24^, our scheme is proposed for optical nonreciprocity with Jaynes-Cummings model in coupled double-cavity system in unbroken ${\mathscr{P}}{\mathscr{T}}$-symmetric phase. However, in^24^ the nonreciprocal transmission is based on the gain-saturation nonlinearity in broken ${\mathscr{P}}{\mathscr{T}}$-symmetric phase. In this case, the weak coupling between the cavities ensures the exponential growth of power in the gain cavity. When the coupling increases and the optical system enters into the unbroken ${\mathscr{P}}{\mathscr{T}}$-symmetric phase, the power will flow fast into the passive cavity and the system becomes linear. A linear system, even with balanced gain and loss, cannot have nonreciprocal response^24^. For our scheme (Fig. 2(b)), the nonreciprocal behaviour can be observed in unbroken ${\mathscr{P}}{\mathscr{T}}$-symmetric phase for Jaynes-Cummings nonlinearity. When the system is in the broken phase or phase transition point, the decreased coupling *J* prevents the flow of power from the passive cavity to the active cavity. The effect of the localization of the field will be reduced considerably and nonreciprocal response will disappear. Similar to^24^, our system is also suitable for optical nonreciprocity with Fig. 5 in broken ${\mathscr{P}}{\mathscr{T}}$-symmetry. When the system is unbroken ${\mathscr{P}}{\mathscr{T}}$-symmetric, the strong coupling *J* will lead to the fast flow of power from the active cavity to the passive cavity. Thus, the effect of field localization will decrease rapidly and nonreciprocal behaviour will fade out.

In summary, we have analyzed the nonreciprocal behaviour with the cavity-cavity coupling hybrid system. The weak Jaynes-Cummings nonlinearity is greatly enhanced by the cavity gain in the active-passive case (i.e., ${\mathscr{P}}{\mathscr{T}}$-symmetric system). With balanced gain and loss, we obtain the non-lossy and high isolation ratio nonreciprocal light propagation in the unbroken ${\mathscr{P}}{\mathscr{T}}$ phase. In contrast to other nonlinear schemes^23–25^, we eliminate the risk of the uncertain output field intensity from optical bistability. In addition, the direction of the nonreciprocal light propagation can be switched by changing the cavity gain and other system parameters. Our work provides a promising route for the realization of the optical nonreciprocity and has potential applications in implementing some essential optical elements like optical diodes and isolator.

## Methods

Based on the Hamiltonians [Eqs (1) and (2)], the Heisenberg-Langevin equations, which describe the evolution of the optical composite system, can be obtained. We focus on the the mean response of the optical system, then the operators are reduced to their expectation values, and the Heisenberg-Langevin equations lead to two groups of nonlinear evolution equations. In this work, our goal is to consider the effect of different directions (i.e., forward and backward incidence) of the input field on the output field. With the use of the mean-field assumption $\langle \hat{m}\hat{n}\rangle =\langle \hat{m}\rangle \langle \hat{n}\rangle$ ^49^, two groups of nonlinear evolution equations of the hybrid system with the forward and backward incidence are given by

7 $$\frac{d{a}_{1}^{L}}{dt}={x}_{1}{a}_{1}^{L}-g{\sigma }_{ge}^{L}-iJ{a}_{2}^{L},$$

8 $$\frac{d{a}_{2}^{L}}{dt}={x}_{2}{a}_{2}^{L}-i\,J{a}_{1}^{L}+\sqrt{{\kappa }_{e}}{\varepsilon }_{p},$$

9 $$\frac{d{\sigma }_{z}^{L}}{dt}=-\gamma ({\sigma }_{z}^{L}+1/2)+g{({a}_{1}^{L})}^{\ast }{\sigma }_{ge}^{L}+g{a}_{1}^{L}{({\sigma }_{ge}^{L})}^{\ast },$$

10 $$\frac{d{\sigma }_{ge}^{L}}{dt}={x}_{3}{\sigma }_{ge}^{L}-2g{a}_{1}^{L}{\sigma }_{z}^{L},$$

for the backward incidence and

11 $$\frac{d{a}_{1}^{R}}{dt}={x}_{1}{a}_{1}^{R}-g{\sigma }_{ge}^{R}-iJ{a}_{2}^{R}+\sqrt{{\kappa }_{e}}{\varepsilon }_{p},$$

12 $$\frac{d{a}_{2}^{R}}{dt}={x}_{2}{a}_{2}^{R}-iJ{a}_{1}^{R},$$

13 $$\frac{d{\sigma }_{z}^{R}}{dt}=-\gamma ({\sigma }_{z}^{R}+1/2)+g{({a}_{1}^{R})}^{\ast }{\sigma }_{ge}^{R}+g{a}_{1}^{R}{({\sigma }_{ge}^{R})}^{\ast },$$

14 $$\frac{d{\sigma }_{ge}^{R}}{dt}={x}_{3}{\sigma }_{ge}^{R}-2g{a}_{1}^{R}{\sigma }_{z}^{R},$$

for the forward incidence. In the above equations, *x*_1_ = −(*i*Δ_1_ + *κ*_1_/2 + *κ*_*e*_/2), *x*_2_ = −(*i*Δ_1_ + *κ*_2_/2 + *κ*_*e*_/2), and *x*_3_ = −*i*(Δ_1_ + Δ_2_) − *γ*/2 with *γ* the spontaneous emission decay rate and *κ*_*j*_ the cavity intrinsic decay rate. ${\hat{\sigma }}_{z}=({\hat{\sigma }}_{ee}-{\hat{\sigma }}_{gg})/2$ denotes the population inversion operator and ${\sigma }_{z}=\langle {\hat{\sigma }}_{z}\rangle$. *κ*_*j*_ > 0 and *κ*_*j*_ < 0 (*j* = 1, 2) correspond to a passive cavity and an active cavity, respectively. The gain cavity can be fabricated with erbium-doped silica film on a silicon wafer. The optical gain is obtained through pumping the erbium ions with a pump laser, whose resonant frequency is different from cavity resonant frequency^24,25^. Thus, the cavity field is only coupled to our emitter rather than the erbium ions because of the large frequency detuning.

To end this part, it is instructive to briefly analyze the principal mechanism behind ${\mathscr{P}}{\mathscr{T}}$- symmetry in our studied system. The Hamiltonian of the coupled active-passive double cavities system can be given by

15 $$\hat{H}=\hslash [{\omega }_{c}-i({\kappa }_{1}+{\kappa }_{e})]{\hat{a}}_{1}^{\dagger }{\hat{a}}_{1}+\hslash [{\omega }_{c}-i({\kappa }_{2}+{\kappa }_{e})]{\hat{a}}_{2}^{\dagger }{\hat{a}}_{2}+\hslash J({\hat{a}}_{2}^{\dagger }{\hat{a}}_{1}+{\hat{a}}_{1}^{\dagger }{\hat{a}}_{2}),$$

As a subsystem of our system, the coupled double cavities have two supermodes and the corresponding eigen frequencies are

16 $${\omega }_{\pm }={\omega }_{c}-\frac{i}{4}({\kappa }_{1}+{\kappa }_{2}+2{\kappa }_{e})\pm \frac{1}{2}\sqrt{4{J}^{2}-\frac{{({\kappa }_{1}-{\kappa }_{2})}^{2}}{4}}$$

with *ω*_1_ = *ω*_2_ = *ω*_*c*_. To balance gain and loss, we set *κ*_1_ = −(*κ*_2_ + 2*κ*_*e*_). When $J > \tfrac{1}{2}({\kappa }_{2}+{\kappa }_{e})$, the system is in the ${\mathscr{P}}{\mathscr{T}}$-symmetric regime, and the supermodes are nondegenerate. The imaginary parts of *ω*_±_ disappear and one can get the real ${\mathscr{P}}{\mathscr{T}}$ spectra ${\omega }_{\pm }={\omega }_{c}\pm \tfrac{1}{2}\sqrt{4{J}^{2}-\tfrac{{({\kappa }_{1}-{\kappa }_{2})}^{2}}{4}}$. In this case, the real energy spectrums with a zero linewidth are displaced at $\pm \tfrac{1}{2}\sqrt{4{J}^{2}-\tfrac{{({\kappa }_{1}-{\kappa }_{2})}^{2}}{4}}$ away from the central frequency *ω*_*c*_. When *J* decreases to $\tfrac{1}{2}({\kappa }_{2}+{\kappa }_{e})$, the two supermodes becomes degenerate and combine into the central frequency *ω*_*c*_. $J=\tfrac{1}{2}({\kappa }_{2}+{\kappa }_{e})$ is actually the ${\mathscr{P}}{\mathscr{T}}$-transition point, i.e., exceptional point. The eigen frequencies will become complex and the ${\mathscr{P}}{\mathscr{T}}$ -symmetric phase is broken once $J < \tfrac{1}{2}({\kappa }_{2}+{\kappa }_{e})$. In the broken ${\mathscr{P}}{\mathscr{T}}$-symmetric regime, one supermode is amplified and the other gradually vanishes because of the absorption. It has been confirmed that the ${\mathscr{P}}{\mathscr{T}}$ phase transition of the subsystem has a significant impact on the dynamics of the full system^24,25,33,39^.

**Figure 6:**
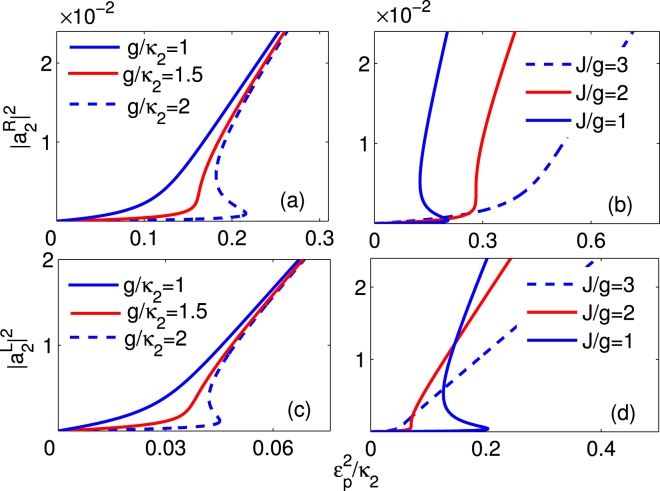
The output field controlled by the coupling strengths. For the passive-passive case, the output field ${|{a}_{2}^{R}|}^{2},\,{|{a}_{1}^{L}|}^{2}$ as a function of the input field ${\varepsilon }_{p}^{2}/{\kappa }_{2}$ with (**a**,**c**) J = 2*κ*_2_ and (**b**,**d**) g = 2*κ*_2_. The other system parameters are chosen as *γ* = 0.1*κ*_2_, Δ_1_ = Δ_2_ = 0, *κ*_1_ = *κ*_2_, and *κ*_*e*_ = 3*κ*_2_, respectively.

**Figure 7:**
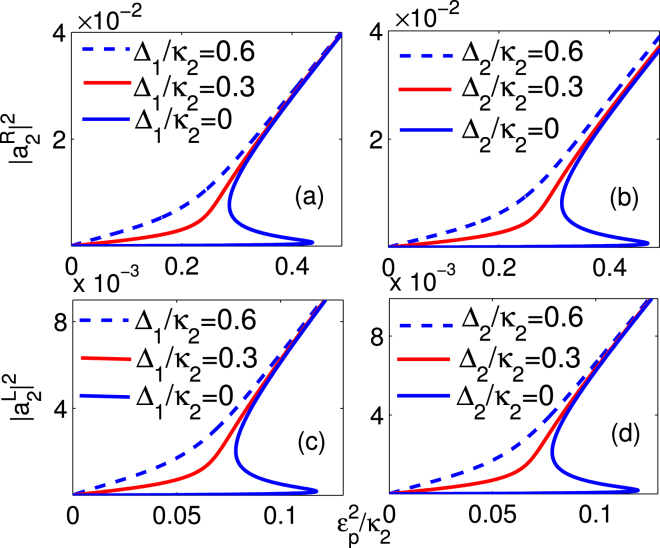
The output field controlled by the frequency detuning. The output field (**a**,**c**) ${|{a}_{2}^{R}|}^{2}$, (**b**,**d**) ${|{a}_{1}^{L}|}^{2}$ as a function of the input field ${\varepsilon }_{p}^{2}/{\kappa }_{2}$ with J = 4*κ*_2_ and g = 2*κ*_2_. The other system parameters are the same as in Fig. 2.

To avoid the negative effect of the inconclusive output field on the nonreciprocal light propagation, we study the optical bistable behaviour carefully. By seeking the numerical steady-state solution of Eqs (7–14), we show that the steady-state output field intensity ${P}_{out}^{R}\propto {|{a}_{2}^{R}|}^{2}$ and ${P}_{out}^{L}\propto {|{a}_{1}^{L}|}^{2}$ versus the input filed intensity ${P}_{in}^{L(R)}\propto {\varepsilon }_{p}^{2}$ under various parametric conditions in Fig. 6. The influence of the cavity-quantum emitter coupling strength *g* on the behaviour of the optical bistability is shown in Fig. 6(a,c). The bistable threshold increases gradually as the strength *g* increases. More importantly, the area of the hysteresis loop becomes broader as the coupling strength *g* increases from 1*κ*_2_ to 2*κ*_2_. Conversely, the optical bistable regions will disappear when the coupling strength *g* is small enough. It can be explained that the optical bistability is caused by the nonlinear terms $g{a}_{1}^{\ast }{\sigma }_{ge}$, $g{a}_{1}{\sigma }_{ge}^{\ast }$ and −2*ga*_1_ *σ*_*z*_, and at a large extent, the nonlinearity of the optical system grows as the coupling strength *g* increases. On the other hand, the cavity-cavity coupling strength *J* has an opposite influence on the optical bistability. As shown in Fig. 6(b,d), there is a competition between the cavity-quantum emitter coupling and the cavity-cavity coupling in the input field of the optical system. When *g* = *J*, there is a remarkable optical bistable region, which quickly becomes narrow as the ratio *J*/*g* increases. Once the cavity-cavity coupling has an overwhelming advantage against the cavity-quantum emitter coupling (i.e., *J* ≥ 3*g*), the nonlinearity of the system can be neglected and the optical bistable area will disappear completely. Besides the cavity-cavity coupling strength *J*, the frequency detunings Δ_*j*_(*j* = 1, 2) also have a significant impact the optical stability. The increasing frequency detuning will weaken the cavity-quantum emitter coupling and makes the optical bistable area small. As shown in Fig. 7, the optical bistable area gradually becomes narrow until disappears as the frequency detuning increases.
